# Formaldehyde and Epigenetic Alterations: MicroRNA Changes in the Nasal Epithelium of Nonhuman Primates

**DOI:** 10.1289/ehp.1205582

**Published:** 2013-01-15

**Authors:** Julia E. Rager, Benjamin C. Moeller, Melanie Doyle-Eisele, Dean Kracko, James A. Swenberg, Rebecca C. Fry

**Affiliations:** 1 Department of Environmental Sciences and Engineering, Gillings School of Global Public Health, and; 2 Curriculum in Toxicology, University of North Carolina at Chapel Hill, Chapel Hill, North Carolina, USA; 3 Lovelace Respiratory Research Institute, Albuquerque, New Mexico, USA; 4 Center for Environmental Health and Susceptibility, Gillings School of Global Public Health, University of North Carolina at Chapel Hill, Chapel Hill, North Carolina, USA

**Keywords:** apoptosis, epigenetics, formaldehyde, microRNA, primate, systems biology

## Abstract

Background: Formaldehyde is an air pollutant present in both indoor and outdoor atmospheres. Because of its ubiquitous nature, it is imperative to understand the mechanisms underlying formaldehyde-induced toxicity and carcinogenicity. MicroRNAs (miRNAs) can influence disease caused by environmental exposures, yet miRNAs are understudied in relation to formaldehyde. Our previous investigation demonstrated that formaldehyde exposure in human lung cells caused disruptions in miRNA expression profiles *in vitro*.

Objectives: Using an *in vivo* model, we set out to test the hypothesis that formaldehyde inhalation exposure significantly alters miRNA expression profiles within the nasal epithelium of nonhuman primates.

Methods: Cynomolgus macaques were exposed by inhalation to approximately 0, 2, or 6 ppm formaldehyde for 6 hr/day for 2 consecutive days. Small RNAs were extracted from nasal samples and assessed for genome-wide miRNA expression levels. Transcriptional targets of formaldehyde-altered miRNAs were computationally predicted, analyzed at the systems level, and assessed using real-time reverse transcriptase polymerase chain reaction (RT-PCR).

Results: Expression analysis revealed that 3 and 13 miRNAs were dysregulated in response to 2 and 6 ppm formaldehyde, respectively. Transcriptional targets of the miRNA with the greatest increase (miR-125b) and decrease (miR-142-3p) in expression were predicted and analyzed at the systems level. Enrichment was identified for miR-125b targeting genes involved in apoptosis signaling. The apoptosis-related targets were functionally tested using RT-PCR, where all targets showed decreased expression in formaldehyde-exposed samples.

Conclusions: Formaldehyde exposure significantly disrupts miRNA expression profiles within the nasal epithelium, and these alterations likely influence apoptosis signaling.

Formaldehyde is a ubiquitous chemical that has been the focus of many toxicological and epidemiological investigations. Epidemiological studies have found that formaldehyde is associated with increased risk of childhood asthma (McGwin et al. 2010), acute respiratory tract illness (Tuthill 1984), sinonasal cancer (Luce et al. 1993), nasopharyngeal cancer (Vaughan et al. 2000), and possibly leukemia (Beane Freeman et al. 2009). In toxicological studies, formaldehyde has been shown to cause nasal squamous cell carcinomas in rats (Kerns et al. 1983; Monticello et al. 1996) and, to a lesser extent, in mice (Kerns et al. 1983). Formaldehyde is currently classified as a known human carcinogen by the International Agency for Research on Cancer (IARC 2006).

Formaldehyde is present in both indoor and outdoor atmospheres. In indoor environments, sources of formaldehyde include plywood, furniture, particle board, certain insulation materials, carpets, paints and varnishes, textiles, tobacco smoke, and the use of formaldehyde as a disinfectant [IARC 2006; National Toxicology Program (NTP) 2011]. In outdoor environments, formaldehyde is produced as both a primary and secondary air pollutant via atmospheric photochemistry (IARC 2006; NTP 2011). Some of the highest formaldehyde exposures occur in occupational settings such as industries involving resin, plastics, wood, paper, insulation, textiles, chemical productions, disinfectants, and embalming products (IARC 2006; NTP 2011). Formaldehyde is also formed *in vivo* through the metabolism and processing of drugs, dietary agents, and amino acids (O’Brien et al. 2005). Because of the constant presence of both endogenous and environmental formaldehyde exposure, coupled with its deleterious health effects, understanding the exposure response and biological basis of formaldehyde-induced health effects is of utmost importance.

A key mode of action that links formaldehyde exposure to cancer involves damage to DNA (Lu et al. 2011; NTP 2011). Formaldehyde is a direct-acting genotoxic compound that induces DNA adducts, DNA–protein crosslinks, DNA–DNA crosslinks, DNA single-strand breaks, and gene mutations in cultured mammalian cells (NTP 2011). Likewise, formaldehyde inhalation exposure *in vivo* has been shown to cause increased DNA adduct formation in nasal tissue from nonhuman primates (Moeller et al. 2011) and rats (Lu et al. 2011). When DNA damage occurs in tumor suppressors or genes regulating the cell cycle, carcinogenesis may occur (Hanahan and Weinberg 2011). Mutations in the *p53* tumor suppressor gene have been demonstrated in formaldehyde-induced nasal squamous cell carcinomas in rats (Recio et al. 1992). Cell proliferation associated with cytotoxicity also plays a key role in formaldehyde carcinogenesis (Chang et al. 1983; NTP 2011). Systems-based analyses employed to understand formaldehyde’s effects on cellular regulation should increase our current understanding of formaldehyde-induced disease.

We investigated possible epigenetic changes caused by formaldehyde exposure in order to test molecular mechanisms potentially underlying formaldehyde-induced health effects. We previously showed that gaseous formaldehyde is capable of significantly disrupting microRNA (miRNA) expression profiles in airway epithelial cells *in vitro* (Rager et al. 2011). With this finding, we proposed that miRNAs may play key roles in formaldehyde-induced effects in various cell types and systems. These small molecules are a part of the epigenetic machinery (Iorio et al. 2010) regulating mRNA abundance and protein production (Friedman et al. 2009). By base pairing to target mRNAs, miRNAs can cause mRNA degradation and/or translational repression (Friedman et al. 2009). Human miRNAs are estimated to regulate more than 60% of all protein-coding genes (Friedman et al. 2009). Because miRNAs play such pivotal roles in gene regulation, it is important to understand the influence formaldehyde exposure may have on miRNA expression signatures.

The present study is the first to investigate potential changes in miRNA expression profiles induced by inhaled formaldehyde exposure *in vivo*. Cynomolgus macaques were exposed to target concentrations of 0, 2, or 6 ppm formaldehyde. These concentrations represent potential occupational exposure levels: Formaldehyde levels up to and greater than 6 ppm have been measured in certain occupational settings including industries related to formaldehyde-based resin production, plastic production, and biology/pathology laboratories (NTP 2011). Formaldehyde concentrations of 2 ppm have also been measured in mobile homes (Salthammer et al. 2010). Genome-wide microarray analysis of small RNA molecules within nasal tissue revealed that miRNA expression profiles were significantly disrupted by formaldehyde. To gain further information on the mechanistic consequences of miRNA changes, transcriptional targets of formaldehyde-responsive miRNAs were predicted and assessed at the systems level. Taken together, this research suggests a novel miRNA-mediated mechanism through which formaldehyde may induce alterations in biological effects.

## Materials and Methods

*Ethics statement.* Cynomolgus macaques were treated humanely and with regard for alleviation of suffering. Animals were exposed, sedated, and euthanized using protocols approved by the Lovelace Research Institute’s animal care and use committee.

*Animals*. Eight male cynomolgus macaques (*Macaca fascicularis*) were selected from the Lovelace Respiratory Research Institute colony. Animals were approximately 6 years of age and weighed between 4.48 and 8.56 kg. Animals were conditioned to whole-body exposure chambers for 30, 60, 180, and 360 min before the first day of exposure, as previously described (Moeller et al. 2011).

*Formaldehyde exposures*. Animals were exposed to formaldehyde over the course of 2 days for 6 hr each day using whole-body exposure chambers. Target exposure concentrations were 0, 2, and 6 ppm formaldehyde. Exposure conditions were created by vaporizing [^13^CD_2_]-paraformaldehyde. Formaldehyde was isotope-labeled for a previous investigation (Moeller et al. 2011). Chamber concentrations were monitored by collecting samples with a Waters XpoSure Aldehyde Sampler cartridge (Milford, MA) every 5 min throughout each exposure period. Samples from the cartridges were analyzed using high-performance liquid chromatography with an attached detector monitoring ultraviolet absorbance at 360 nm (Lu et al. 2011; Moeller et al. 2011). Two control animals were placed in whole-body exposure chambers containing clean air. Three nonhuman primates were exposed to a target concentration of 2 ppm formaldehyde, where the measured concentration averaged 1.9 ppm across the exposure periods. Three nonhuman primates were exposed to a target concentration of 6 ppm formaldehyde, where the measured concentration averaged 6.1 ppm across the exposure periods. For more detailed methods, see Moeller et al. (2011).

*Sample collection*. Approximately 15 min after the second exposure period, animals were serially sedated with Ketamine (10 mg/kg, intramuscular) and euthanized with Euthasol (> 1 mL/4.5 kg, intravenous). Animals underwent necropsy one at a time with each necropsy requiring approximately 45 min. All samples were collected within 3 hr of the exposure. Sample collection started immediately after the last exposure in order to parallel sacrifice and sample collection times used in our previous studies (Lu et al. 2011; Moeller et al. 2011). During necropsy, nasal epithelial tissue from the maxilloturbinate regions were collected, placed in RNAlater® RNA stabilization reagent (Qiagen, Valencia, CA), and stored at –80°C. Samples were shipped by overnight courier on dry ice to the University of North Carolina at Chapel Hill.

*Sample processing*. Small RNAs were isolated from nasal tissue samples. First we disrupted and homogenized the samples using a TissueRuptor (Qiagen) in the presence of TRIzol (Invitrogen Life Technologies, Carlsbad, CA). We then isolated RNA using the miRNeasy® kit (Qiagen). Extracted RNA was quantified with a Nanodrop 1000 spectrophotometer (Thermo Scientific, Waltham, MA) and its integrity verified with a 2100 Bioanalyzer (Agilent Technologies, Santa Clara, CA). RNA was then labeled and hybridized to the Agilent Human miRNA Microarray (v1.0). This microarray assesses the relative expression levels of 534 miRNAs measured using 11,080 probesets. Microarray results were extracted using Agilent Feature Extraction software. Microarray data have been submitted to the National Center for Biotechnology Information (NCBI) Gene Expression Omnibus repository (Edgar et al. 2002) and are available under accession number GSE34978 (NCBI 2010).

*Microarray analysis*. Microarray data were normalized by quantile normalization. To eliminate background noise, miRNA probes with signal intensities < 40 (i.e., the median signal) across all replicates were removed. Differential expression was defined as a significant difference in miRNA levels between exposed versus unexposed samples, where three statistical requirements were set: *a*) fold change of ≥ 1.5 or ≤ –1.5 (average exposed vs. average unexposed); *b*) *p*-value < 0.05 [analysis of variance (ANOVA)]; and *c*) a false discovery rate corrected *q*-value < 0.1. ANOVA *p*-values were calculated using Partek® Genomics Suite™ software (St. Louis, MO). To control the rate of false positives, *q*-values were calculated as the minimum “positive false discovery rate” that can occur when identifying significant hypotheses (Storey 2003).

*Real-time reverse transcriptase polymerase chain reaction (RT-PCR) confirmation of miRNA expression changes*. To confirm formaldehyde-induced miRNA expression changes, we performed RT-PCR using two miRNAs identified as the most increased in expression (miR-125b and miR-152) and the two miRNAs identified as the most decreased in expression (miR-145 and miR-142-3p) after 6 ppm formaldehyde exposure. TaqMan® MicroRNA Primer Assays for hsa-miR-125b (ID 000449), hsa-miR-152 (ID 000475), hsa-miR-145 (ID 002278), and hsa-miR-142-3p (ID 000464) were used in conjunction with the TaqMan® Small RNA Assays PCR kit (Applied Biosystems, Carlsbad, CA). The same control and formaldehyde-exposed samples from the microarray analysis were used for RT-PCR, and samples were assessed in technical triplicate. The resulting RT-PCR cycle times were normalized against the U6 housekeeping miRNA, and fold changes in expression were calculated using the ΔΔCt method. Statistical significance of the difference in miRNA expression levels between the formaldehyde-exposed and unexposed samples was calculated using an ANOVA (Partek®).

*Predicting targets of miR-125b and miR-142-3p*. We carried out computational predictions of the mRNA targets of miR-125b and miR-142-3 in order to understand the impact of formaldehyde-responsive miRNAs on gene expression levels. These two miRNAs were selected because they showed the largest increase (miR-125b) or decrease (miR-142-3p) in expression after 6 ppm formaldehyde exposure. Here, TargetScanHuman release 5.2 (Whitehead Institute for Biomedical Research, Cambridge, MA) algorithms were employed to identify potential matches between 3´ untranslated mRNA regions and miRNA seed sequences (Lewis et al. 2005). The resulting predicted miRNA × mRNA interactions were filtered for the probability of preferentially conserved targeting (P_CT_) ≥ 0.9. This P_CT_ filter controlled for background conservation across mammals by accounting for mutational biases, dinucleotide conservation rates, and individual untranslated region conservation rates (Friedman et al. 2009).

*Pathway enrichment analysis of predicted targets*. Network analysis was performed to understand the systems-level response to formaldehyde inhalation exposure possibly mediated via epigenetic (e.g., miRNA) regulation. For this analysis, the predicted mRNA targets of miR-125b and miR-142-3p were overlaid onto a global interaction network. Networks were algorithmically constructed based on connectivity, as enabled through Ingenuity Pathway Analysis (Ingenuity Systems, Redwood City, CA). Canonical pathways within the constructed networks were then identified. Overrepresented pathways were defined as pathways than contain more targets than expected by chance, as calculated using the right-tailed Fisher’s exact test. Pathways with enrichment *p*-values < 0.05 were considered significantly enriched with the predicted targets of miR-125b or miR-142-3p.

*Testing miRNA targets using RT-PCR*. All apoptosis-associated genes (*n* = 4) predicted to be regulated by formaldehyde-responsive miR-125b, and all integrin-linked kinase (ILK)-associated genes (*n* = 2) predicted to be regulated by formaldehyde-responsive miR-142-3p, were tested at the gene expression level using RT-PCR. QuantiTect Primer Assays were used with QuantiTect SYBR® Green PCR kits (Qiagen) and the LightCycler® 480 (Roche Applied Science, Indianapolis, IN). Specifically, BCL2-antagonist/killer 1 (*BAK1*; catalog Number QT00228508), caspase 2, apoptosis-related cysteine peptidase (*CASP2*; QT01342509), integrin, beta 8 (*ITGB8*; QT00038507), mitogen-activated protein kinase kinase 7 (*MAP2K7*; QT00090545), myeloid cell leukemia sequence 1 (BCL2-related) (*MCL1*; QT00094122), and the rapamycin-insensitive companion of mTOR (*RICTOR*; QT00065793) were evaluated for potential changes in gene expression levels induced by formaldehyde exposure. Resulting RT-PCR cycle times were normalized against the β-actin housekeeping gene, and fold changes in expression were calculated using the ΔΔCt method. Statistical significance comparing the expression levels between exposed and unexposed samples was calculated using an ANOVA (Partek®).

## Results

*Formaldehyde disrupts miRNA expression profiles in nasal tissue*. To study the effects of formaldehyde inhalation exposure, cynomolgus macaques were exposed to approximately 0, 2, or 6 ppm formaldehyde 6 hr/day for 2 days. After treatment, nasal epithelial tissue samples were collected and assessed for genome-wide changes in miRNA expression profiles using the Agilent Human miRNA Microarray. Microarray analysis identified 3 miRNAs with significantly decreased expression levels upon exposure to 2 ppm formaldehyde (Table 1). In comparison, exposure to 6 ppm formaldehyde significantly disrupted the expression levels of 13 miRNAs, represented by 15 array probesets (Table 1). Of the 13 miRNAs, 4 were significantly increased and 9 were significantly decreased in expression. Interestingly, the three miRNAs that were significantly decreased in response to 2 ppm formaldehyde (i.e., miR-142-3p, miR-145, and miR-203) were also significantly decreased in response to 6 ppm formaldehyde.

**Table 1:** Formaldehyde inhalation exposure in nonhuman primates significantly disrupts the expression levels of 13 unique miRNAs, represented by 15 array probesets.

miRNA	Agilent array feature number	2 ppm formaldehyde			6 ppm formaldehyde		
		log2FC	p-Value	q-Value	log2FC	p-Value	q-Value
miR-125b	2637	0.44	6.1 × 10–1	0.666	2.86*	2.2 × 10–4	0.090
miR-152	1548	0.79	3.0 × 10–3	0.297	1.29*	1.3 × 10–4	0.072
miR-219-5p	1180	0.36	8.8 × 10–2	0.451	1.22*	1.7 × 10–4	0.075
miR-532-5p	1259	0.35	3.4 × 10–2	0.390	1.09*	8.1 × 10–5	0.055
miR-520f	14457	–0.61	3.3 × 10–4	0.188	–0.77*	1.4 × 10–4	0.072
miR-26b	12607	–1.13	9.3 × 10–5	0.146	–1.38*	5.2 × 10–5	0.050
miR-140-5p	12026	–0.69	3.6 × 10–4	0.188	–1.56*	2.4 × 10–5	0.036
miR-22	12927	–0.69	4.8 × 10–4	0.203	–1.70*	2.6 × 10–5	0.036
miR-374a	14431	–1.68	1.2 × 10–4	0.148	–1.77*	1.1 × 10–4	0.067
miR-203	12162	–1.98*	4.7 × 10–5	0.098	–2.11*	4.1 × 10–5	0.046
miR-203	11451	–1.75	1.0 × 10–4	0.146	–2.12*	6.7 × 10–5	0.055
miR-142-3p	12366	–4.12*	1.1 × 10–6	0.009	–2.92*	1.6 × 10–6	0.011
miR-29a	13448	–3.24	2.5 × 10–4	0.188	–3.15*	2.6 × 10–4	0.099
miR-145	15649	–3.15*	3.0 × 10–5	0.098	–3.56*	2.6 × 10–5	0.036
miR-142-3p	14658	–2.81	3.1 × 10–4	0.188	–5.01*	1.8 × 10–4	0.075
FC, fold change. *p < 0.01, q < 0.1 for FC relative to unexposed samples.							

**Figure 1:**
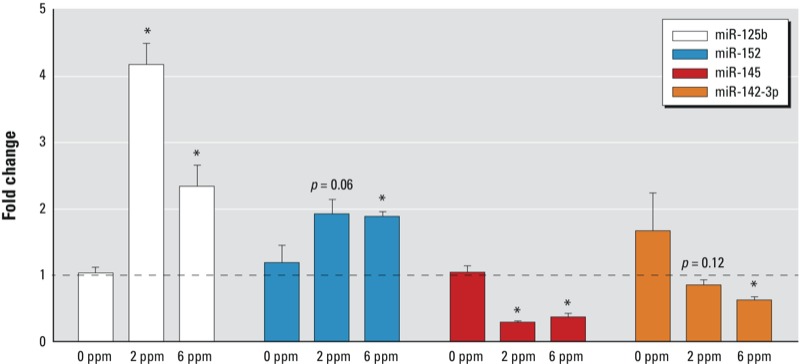
RT-PCR confirms the altered expression of selected miRNAs upon exposure to formaldehyde. Data are presented as mean fold changes (exposed/unexposed) (± SE) in gene expression. *p < 0.05 compared with 0 ppm control.

*RT-PCR confirmed formaldehyde-induced miRNA expression changes*. RT-PCR was performed to confirm that formaldehyde inhalation exposure significantly disrupts the expression of miRNAs. Specifically, the two miRNAs most increased in expression (miR-125b and miR-152) and the two miRNAs most decreased in expression (miR-145 and miR-142-3p) in response to 6 ppm formaldehyde were validated using this alternative method. Comparing the exposed versus unexposed samples confirmed that miR-125b and miR-152, were, indeed, significantly (*p* < 0.05) increased in expression upon exposure to 6 ppm formaldehyde (Figure 1). The microarray analysis’ stringent multiple test correction filter excluded miR-125b from the list of miRNAs significantly differentially expressed by 2 ppm formaldehyde. However, RT-PCR analysis showed that miR-125b was significantly increased in expression in the 2 ppm formaldehyde-exposed animals. Similar confirmation was observed for miR-145 and miR-142-3p, where expression levels were significantly (*p* < 0.05) decreased following 6 ppm formaldehyde exposure (Figure 1). Microarray analysis also showed that the expression level of miR-145 was significantly decreased upon exposure to 2 ppm formaldehyde. This change in expression was verified with RT-PCR (Figure 1).

*Transcriptional targets of miR-125b and miR-142-3p were predicted*. To understand genomic changes regulated via miRNAs that formaldehyde inhalation exposure may initiate, we computationally predicted mRNA targets of miR-125b and miR-142-3p. These miRNAs were selected for further investigation because they showed the highest increase or decrease in expression upon exposure to 6 ppm formaldehyde, respectively. In addition, their differential expression was confirmed through RT-PCR analysis. Using seed match–based algorithms, a total of 132 genes were predicted to be targeted by miR-125b [see Supplemental Material, Table S1 (http://dx.doi.org/10.1289/ehp.1205582)]. In comparison, only 13 genes were predicted to be targeted by miR-142-3p (see Supplemental Material, Table S2).

*Apoptosis signaling is associated with miR-125b predicted targets*. Enriched canonical signaling pathways were evaluated for the 132 predicted targets of miR-125b in order to evaluate the potential effects of formaldehyde exposure at the systems level. Through this network analysis, 11 canonical pathways were identified as significantly overrepresented amongst the networks constructed using the predicted targets of miR-125b (Table 2). The two pathways of highest significance were sphingolipid metabolism (*p* = 0.003) and apoptosis signaling (*p* = 0.003) [Table 2, see also Supplemental Material, Figure S1 (http://dx.doi.org/10.1289/ehp.1205582)].

**Table 2:** Pathways significantly associated with the predicted targets of miR-125b.

Canonical pathways	p-Value	miR-125b predicted targets
Sphingolipid metabolism	0.003	ACER2, FUT4, NEU1, SGPL1
Apoptosis signaling	0.003	BAK1, CASP2, MAP2K7, MCL1
Glycosphingolipid biosynthesis—globoseries	0.012	FUT4, ST8SIA4
Glycosphingolipid biosynthesis—neolactoseries	0.012	FUT4, ST8SIA4
Glycosphingolipid biosynthesis—ganglioseries	0.014	FUT4, ST8SIA4
N-glycan degradation	0.014	MAN1B1, NEU1
O-glycan biosynthesis	0.017	FUT4, GCNT1
N-glycan biosynthesis	0.037	FUT4, MAN1B1
Sphingosine-1-phosphate signaling	0.039	ACER2, CASP2, RND2
TNFR1	0.042	CASP2, TNFAIP3
Semaphorin signaling in neurons	0.048	RND2, SEMA4D
Abbreviations: ACER2, alkaline ceramidase 2; FUT4, fucosyltransferase 4; GCNT1, glucosaminyl (N-acetyl) transferase 1, core 2; MAN1B1, mannosidase, alpha, class 1B, member 1; NEU1, sialidase 1 (lysosomal sialidase); RND2, Rho family GTPase 2; SEMA4D, sema domain, immunoglobulin domain (Ig), transmembrane domain (TM) and short cytoplasmic domain, (semaphorin) 4D; SGPL1, sphingosine-1-phosphate lyase 1; ST8SIA4, ST8 alpha-N-acetyl-neuraminide alpha-2,8-sialyltransferase 4; TNFAIP3, tumor necrosis factor, alpha-induced protein 3; TNFR1, tumor necrosis factor binding protein 1.		

**Figure 2:**
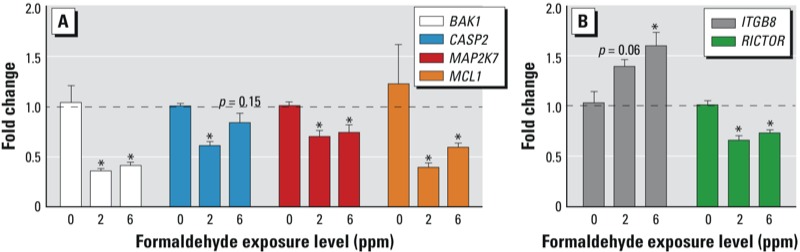
RT-PCR shows the altered expression of (*A*) apoptosis signaling-related genes predicted to be targeted by miR-125b, and (*B*) ILK signaling-related genes predicted to be targeted by miR-142-3p. Data are presented as mean fold changes (exposed/unexposed) (± SE) in gene expression. *p < 0.05 compared with 0 ppm control.

*Apoptosis-related miR-125b targets are decreased in expression*. All four of the apoptosis-related mRNA molecules predicted to be targeted by miR-125b were tested at the gene expression level using RT-PCR. As miR-125b was increased in expression, it was anticipated that its potential targets would be decreased in expression after formaldehyde exposure. Three of the evaluated targets, *BAK1*, *MAP2K7*, and *MCL1*, showed significantly (*p* < 0.05) decreased expression levels in response to both 2 and 6 ppm formaldehyde exposures (Figure 2A). *CASP2* showed significantly decreased expression in response to 2 ppm formaldehyde. *CASP2* expression was also decreased in response to 6 ppm formaldehyde, but was not statistically significant (*p* = 0.15) (Figure 2A). Altogether, all four of the apoptosis-related mRNAs predicted to be regulated by miR-125b showed decreased expression upon exposure to formaldehyde.

*ILK signaling is associated with miR-142-3p predicted targets*. To further assess the potential effects of formaldehyde exposure at the systems level, enriched canonical signaling pathways were evaluated for the 13 predicted targets of miR-142-3p. Three canonical pathways were identified as significantly overrepresented within the predicted targets of miR-142-3p [see Supplemental Material, Table S3 (http://dx.doi.org/10.1289/ehp.1205582)]. The pathway of highest significance was ILK signaling (*p* = 0.008).

*ILK-related miR-142-3p targets are altered in expression*. The two ILK signaling–related mRNA molecules predicted to be targeted by miR-142-3p were tested at the gene expression level using RT-PCR. Because miR-142-3p was decreased in expression, it was anticipated that its potential targets would have increased expression after formaldehyde exposure. One of the evaluated targets, *ITGB8*, showed significantly increased expression in response to 6 ppm formaldehyde exposure (Figure 2B). Transcript levels for the other predicted target, *RICTOR*, were significantly decreased in response to 2 and 6 ppm formaldehyde exposure (Figure 2B).

## Discussion

This study is the first to evaluate formaldehyde’s influence on miRNA expression signatures *in vivo*. In order to study the effects of formaldehyde inhalation exposure, nonhuman primates (cynomolgus macaques) were exposed for 6 hr/day over a course of 2 days to approximately 0, 2, or 6 ppm formaldehyde. These exposure levels were selected based on previous investigations showing that exposure to 2 and 6 ppm formaldehyde caused DNA–protein crosslinks (Casanova et al. 1991) and DNA adducts (Moeller et al. 2011) to form within the nasal mucosa of nonhuman primates. The use of nonhuman primates as our animal model is advantageous because the nasal gross anatomy and pattern of airflow are similar between nonhuman primates and humans (Harkema et al. 2006). Furthermore, there is an extremely high degree of similarity in DNA coding and non-coding sequences between macaques and humans (Walker 2008).

After exposure, animals were euthanized, and nasal epithelial samples from the maxilloturbinate region were collected and assessed for genome-wide changes in miRNA expression profiles. Samples from the maxilloturbinate region were used because inhaled formaldehyde is maximally absorbed within this region (Kepler et al. 1998). In addition, our previous investigation revealed that cynomolgus macaques exposed to isotope-labeled [^13^CD_2_]-formaldehyde showed detectable amounts of exogenous (i.e., induced by formaldehyde exposure) and endogenous DNA adducts within nasal samples collected from the maxilloturbinate (Moeller et al. 2011). Specifically, 0.26 ± 0.04 and 0.41 ± 0.05 exogenous *N*^2^-hydroxymethyl-dG/10^7^ dG were present in nonhuman primates exposed to approximately 2 and 6 ppm formaldehyde, respectively (Moeller et al. 2011), and 2.05 ± 0.53 and 2.49 ± 0.39 endogenous *N*^2^-hydroxymethyl-dG/10^7^ dG adducts were present. Furthermore, the respiratory nasal turbinate region of rats exposed to formaldehyde is a site of squamous cell carcinoma formation (Kerns et al. 1983; Monticello et al. 1996).

We measured the expression levels of > 500 miRNAs across two unexposed, three 2 ppm formaldehyde-exposed, and three 6 ppm formaldehyde-exposed nonhuman primates. Although this sample size was robust enough to detect formaldehyde-responsive miRNAs, we recognize that the sample size may have limited the power to detect additional changes in miRNA expression. For the genome-wide analysis, a human miRNA microarray was used because a miRNA microarray is not currently available for nonhuman primates. This array is suitable for these experimental purposes based on the high degree of similarity in DNA sequences, as well as conserved basal gene expression profiles, between humans and cynomolgus macaques (Walker 2008). Baseline human miRNA expression patterns have even been shown to correlate well with cynomolgus macaque miRNA patterns using human miRNA microarrays (Montag et al. 2009). Furthermore, a previous study compared miRNAs identified in the rhesus macaque (*Macaca mulatta*) genome to human homologs and found that 38% of the miRNAs showed 100% homology in precursor sequences (Yue et al. 2008). The remaining 62% of the miRNAs showed between 90% and 100% sequence homology (Yue et al. 2008). Nevertheless, we recognize that certain cynomolgus macaque–specific miRNAs may not be accounted for in these analyses. This results in the potential for an underestimation of formaldehyde’s true impact on genome-wide miRNA profiles in this study. Despite these potential limitations, a set of 13 miRNAs with significant differential expression upon exposure to 2 and/or 6 ppm formaldehyde were identified.

Two of the 13 formaldehyde-responsive miRNAs were among those that we previously showed as altered *in vitro* by formaldehyde, namely miR-26b and miR-140-5p (Rager et al. 2011). This overlap in response suggests that *in vitro* models may show some responses in common with *in vivo* models at the miRNA level. Many of the formaldehyde-responsive miRNAs in the nonhuman primate have known relationships to cancer, where 6 of the 13 formaldehyde-responsive miRNAs have been identified as differentially expressed in human nasopharyngeal carcinoma. More specifically, miR-142-3p, miR-145, miR-152, miR-203, miR-26b, and miR-29a have all been shown to have altered expression levels in nasopharyngeal cancer tissue in comparison to noncancerous tissue (Chen et al. 2009; Li et al. 2011; Sengupta et al. 2008; Wong et al. 2012).

In order to evaluate the effects of formaldehyde inhalation exposure at the systems level, molecular targets of miR-125b and miR-142-3p were computationally predicted and analyzed for pathway enrichment. We focused our systems-based analysis on miR-125b and miR-142-3p because these miRNAs showed the highest increase and decrease in expression, respectively, upon exposure to 6 ppm formaldehyde through microarray analysis and were confirmed using RT-PCR analysis. A total of 132 genes were predicted to be targeted by miR-125b, and thereby decreased at the expression level. Far fewer genes were identified for miR-142-3p, where 13 genes were predicted to be targeted by miR-142-3p, and thereby increased at the expression level.

Canonical pathway enrichment analysis revealed a significant association between the predicted targets of miR-125b and apoptosis signaling. To further test this finding, we evaluated the gene expression levels of all four apoptosis signaling–related genes predicted to be targeted by miR-125b, namely *BAK1*, *CASP2*, *MAP2K7*, and *MCL1*. As predicted, all four genes showed decreased expression levels in the formaldehyde-exposed versus unexposed samples. Two of the apoptosis-related genes predicted to be regulated by miR-125b, *MAP2K7,* and *MCL1* have also been shown to have significantly altered expression levels in the nasal epithelium of rats exposed to formaldehyde (Andersen et al. 2010).

The observed decreased expression of genes involved in apoptosis signaling suggests a possible link between formaldehyde exposure and altered regulation of cell death. For example, BAK1 and CASP2 are both pro-apoptotic and have been shown to induce apoptosis *in vitro* and *in vivo* in several cell types (Kumar 2009; Pataer et al. 2000). While the evaluation of proteins encoded by the apoptosis-related genes would further support these findings, such an assessment was not possible here because proteins were not collected. Still, a similar finding has been observed in the nasal epithelium of rats, where nasal instillation of liquid formaldehyde decreased the expression levels of pro-apoptotic genes (Hester et al. 2003). These findings are of high interest because impaired apoptosis can lead to cellular transformation and cancer development (Hanahan and Weinberg 2011).

Other pathways were also identified as enriched among the predicted targets of miR-125b, including sphingolipid metabolism. Sphingolipids are an abundant class of lipids present at high levels within eukaryotic membranes (Bartke and Hannun 2009). Although sphingolipids were first recognized for their structural roles in membrane formation, more recent work has shown that sphingolipid metabolites are involved in the regulatory signaling of various biological processes, including apoptosis, cell cycle arrest, inflammation, necrosis, and senescence (Bartke and Hannun 2009).

Pathway analysis of the predicted targets of miR-142-3p revealed an enrichment for ILK signaling. It is important to note that this enrichment was not as significant as the enrichment between miR-125b and apoptosis signaling. ILK signaling is involved in a variety of processes within epithelial cells, including cell survival, cell proliferation, and cell adhesion to the extracellular matrix (Gilcrease 2007).

To test our prediction that formaldehyde alters ILK signaling, the expression levels of genes involved in ILK signaling were assessed, including *ITGB8* and *RICTOR*. Because miR-142-3p was decreased in expression by 6 ppm formaldehyde, we anticipated its potential targets to show increased expression. As anticipated, *ITGB8* showed significantly increased expression after 6 ppm formaldehyde exposure. *ITGB8* has been implicated in several biological processes, including airway epithelial cell proliferation (Fjellbirkeland et al. 2003) and airway remodeling involving the extracellular matrix (Kitamura et al. 2011). One of the predicted targets, *RICTOR*, did not show increased transcript levels in formaldehyde-exposed samples. This finding suggests that *a*) miR-142-3p may not influence *RICTOR* under the tested conditions, *b*) miR-142-3p may influence RICTOR protein levels by blocking *RICTOR* translation, or *c*) other mechanisms besides miRNA regulation may influence *RICTOR* expression. Some of these scenarios are supported in a recent study where miRNAs were computationally predicted to target hepatic nuclear factor 4α (*HNF4*α) (Ramamoorthy et al. 2012). The previous research demonstrated that many of the tested miRNAs successfully targeted *HNF4*α. In addition, some of the miRNAs targeted *HNF4*α by blocking *HNF4*α translation, causing the reduced expression of HNF4α protein while leaving transcript levels unchanged (Ramamoorthy et al. 2012).

It is important to note that these results do not demonstrate that miR-125b directly decreases the expression of *BAK1*, *CASP2*, *MAP2K7*, and *MCL1* upon exposure to formaldehyde, nor that miR-142-3p directly increases the expression of *ITGB8*. Indeed, this would be difficult to demonstrate *in vivo*. Rather, we show that formaldehyde is associated with the increased expression of miR-125b and the decreased expression of miR-142-3p, and decreased or increased expression of their respective target genes. However, other studies have confirmed some of these specific miRNA × mRNA interactions. For example, miR-125b has been shown to directly target *BAK1* and downregulate its expression in prostate cancer cells (Shi et al. 2007) and breast cancer cells (Zhou et al. 2010). Our study thereby employed bioinformatics-based approaches to increase knowledge on the interplay between exposure responses, epigenetics, and signaling pathways.

## Conclusions

The present study demonstrates that formaldehyde inhalation exposure significantly disrupts miRNA expression profiles within the nasal epithelium *in vivo*. Systems-level analysis of the transcriptional targets predicted to be regulated by formaldehyde-responsive miR-125b and miR-142-3p revealed the highest enrichment between genes involved in apoptosis signaling and miR-125b. Apoptosis-related gene targets of miR-125b were functionally validated, showing altered transcriptional levels after exposure to formaldehyde in the nasal epithelium. These results provide evidence for a relationship between formaldehyde exposure and altered signaling of the apoptotic machinery, likely regulated via epigenetic mechanisms. These changes in apoptosis-related signaling are of high importance because an inappropriate balance between cell death and survival heavily influences cellular disease state. Future research will compare these changes to potential formaldehyde-induced changes occurring in tissues collected from sites distal to the respiratory tract *in vivo.* These comparisons may provide key information related to the pathophysiological mechanisms of action of formaldehyde.

## Supplemental Material

(696 KB) PDF
