# Supplementary material for: Formaldehyde and Epigenetic Alterations: MicroRNA Changes in the Nasal Epithelium of Nonhuman Primates
Source: Environ Health Perspect. 2013 Jan 15;121(3):339–44. doi: 10.1289/ehp.1205582 (PMC3621188; doi:10.1289/ehp.1205582)

Environmental Health Perspectives doi:10.1289/ehp.1205582

Supplemental Material

Formaldehyde and Epigenetic Alterations: MicroRNA Changes in the Nasal Epithelium of Nonhuman Primates

Julia E. Rager,[1] Benjamin C. Moeller,[2] Melanie Doyle-Eisele,[3] Dean Kracko,[3]
James A. Swenberg,[1,2,4] and Rebecca C. Fry[1,2,*]

[1] Department of Environmental Sciences and Engineering, Gillings School of Global Public Health, The University of North Carolina, Chapel Hill, North Carolina, USA

[2] Curriculum in Toxicology, The University of North Carolina, Chapel Hill, North Carolina, USA

[3] Lovelace Respiratory Research Institute, Albuquerque, New Mexico, USA

[4] Center for Environmental Health and Susceptibility, Gillings School of Global Public Health, The University of North Carolina, Chapel Hill, North Carolina, USA.

Supplemental Material

Table of Contents

Supplemental Material, Table S1: Transcriptional targets predicted to be regulated by miR-125b... **3**

Supplemental Material, Table S2: Transcriptional targets predicted to be regulated by miR-142-3p **7**

Supplemental Material, Table S3: Pathways significantly associated with the predicted targets of miR-142-3p.. **8**

Supplemental Material, Figure S1: Predicted mRNA targets of formaldehyde-altered miR-125b are involved in apoptosis signaling.. .... **9**

Supplemental Material, Table S1: Transcriptional targets predicted to be regulated by miR-125b. P_{CT} refers to the probability of preferentially conserved targeting.

Target	Full Name	Context Score	P_{CT}
GCNT1	glucosaminyl (N-acetyl) transferase 1, core 2 (beta-1,6-N-acetylglucosaminyltransferase)	-0.67	> 0.99
TMEM86A	transmembrane protein 86A	-0.26	> 0.99
PODXL	podocalyxin-like	-0.47	> 0.99
ARID3B	AT rich interactive domain 3B (BRIGHT-like)	-0.51	> 0.99
FLJ20309	hypothetical protein FLJ20309	-0.37	0.99
SH3TC2	SH3 domain and tetratricopeptide repeats 2	-0.73	0.99
PHF15	PHD finger protein 15	-0.31	0.98
GJC1	gap junction protein, gamma 1, 45kDa	-0.37	0.98
KLF13	Kruppel-like factor 13	-0.51	0.97
OLFML2A	olfactomedin-like 2A	-0.49	0.97
MFHAS1	malignant fibrous histiocytoma amplified sequence 1	-0.52	0.97
IRF4	interferon regulatory factor 4	-0.58	0.97
RAPGEF5	Rap guanine nucleotide exchange factor (GEF) 5	-0.39	0.97
LBH	limb bud and heart development homolog (mouse)	-0.37	0.97
ENPP1	ectonucleotide pyrophosphatase/phosphodiesterase 1	-0.4	0.97
UBN1	ubinuclein 1	-0.33	0.97
FAM176A	family with sequence similarity 176, member A	-0.33	0.97
MXD4	MAX dimerization protein 4	-0.07	0.97
SMURF1	SMAD specific E3 ubiquitin protein ligase 1	-0.24	0.97
TLE3	transducin-like enhancer of split 3 (E(sp1) homolog, Drosophila)	-0.2	0.97
TRPS1	trichorhinophalangeal syndrome I	-0.19	0.97
KIAA1522	KIAA1522	-0.37	0.97
ASAH3L	N-acylsphingosine amidohydrolase 3-like	-0.34	0.97
UBE2R2	ubiquitin-conjugating enzyme E2R 2	-0.36	0.97
SEMA4D	sema domain, immunoglobulin domain (Ig), transmembrane domain (TM) and short cytoplasmic domain, (semaphorin) 4D	-0.42	0.97
LIN28	lin-28 homolog (C. elegans)	-0.27	0.97
CPSF6	cleavage and polyadenylation specific factor 6, 68kDa	-0.34	0.97
TGOLN2	trans-golgi network protein 2	-0.39	0.97
OSBPL9	oxysterol binding protein-like 9	-0.45	0.97
TBC1D1	TBC1 (tre-2/USP6, BUB2, cdc16) domain family, member 1	-0.36	0.97
SLC39A9	solute carrier family 39 (zinc transporter), member 9	-0.47	0.96
ENPEP	glutamyl aminopeptidase (aminopeptidase A)	-0.52	0.96
ST8SIA4	ST8 alpha-N-acetyl-neuraminide alpha-2,8-sialyltransferase 4	-0.26	0.96
GRB10	growth factor receptor-bound protein 10	-0.43	0.96
MYT1	myelin transcription factor 1	-0.39	0.96

Target	Full Name	Context Score	P_{CT}
STARD13	StAR-related lipid transfer (START) domain containing 13	-0.99	0.96
PTAR1	protein prenyltransferase alpha subunit repeat containing 1	-0.26	0.96
BMF	Bcl2 modifying factor	-0.38	0.96
ZNRF3	zinc and ring finger 3	-0.24	0.96
ZSCAN29	zinc finger and SCAN domain containing 29	-0.58	0.96
SPTB	spectrin, beta, erythrocytic (includes spherocytosis, clinical type I)	-0.29	0.96
NIN	ninein (GSK3B interacting protein)	-0.38	0.96
SMG1	PI-3-kinase-related kinase SMG-1	-0.31	0.96
NUP210	nucleoporin 210kDa	-0.55	0.96
GGA2	golgi associated, gamma adaptin ear containing, ARF binding protein 2	-0.31	0.96
DUS1L	dihydrouridine synthase 1-like (S. cerevisiae)	-0.48	0.96
MTF1	metal-regulatory transcription factor 1	-0.33	0.96
C14orf43	chromosome 14 open reading frame 43	-0.13	0.96
PPAT	phosphoribosyl pyrophosphate amidotransferase	-0.43	0.96
SMEK1	SMEK homolog 1, suppressor of mek1 (Dictyostelium)	-0.61	0.96
ORC2L	origin recognition complex, subunit 2-like (yeast)	-0.33	0.96
ACHE	acetylcholinesterase (Yt blood group)	-0.57	0.96
CGN	cingulin	-0.43	0.95
LRP4	low density lipoprotein receptor-related protein 4	-0.28	0.95
C6orf47	chromosome 6 open reading frame 47	-0.3	0.95
TMEM77	transmembrane protein 77	-0.5	0.95
KCNA1	potassium voltage-gated channel, shaker-related subfamily, member 1 (episodic ataxia with myokymia)	-0.24	0.95
KIAA0174	KIAA0174	-0.29	0.95
LFNG	LFNG O-fucosylpeptide 3-beta-N-acetylglucosaminyltransferase	-0.48	0.95
HIC2	hypermethylated in cancer 2	-0.02	0.95
TNFAIP3	tumor necrosis factor, alpha-induced protein 3	-0.29	0.95
CASP2	caspase 2, apoptosis-related cysteine peptidase (neural precursor cell expressed, developmentally down-regulated 2)	-0.25	0.95
CRB2	crumbs homolog 2 (Drosophila)	-0.44	0.95
KIAA0317	KIAA0317	-0.13	0.95
SCARB1	scavenger receptor class B, member 1	-0.34	0.95
ANPEP	alanyl (membrane) aminopeptidase (aminopeptidase N, aminopeptidase M, microsomal aminopeptidase, CD13, p150)	-0.3	0.95
PSTPIP2	proline-serine-threonine phosphatase interacting protein 2	-0.39	0.95
CYP24A1	cytochrome P450, family 24, subfamily A, polypeptide 1	-0.41	0.95
ZSWIM5	zinc finger, SWIM-type containing 5	-0.4	0.95

Target	Full Name	Context Score	P_{CT}
NCAN	neurocan	-0.23	0.95
TNFSF4	tumor necrosis factor (ligand) superfamily, member 4 (tax-transcriptionally activated glycoprotein 1, 34kDa)	-0.49	0.95
NECAB3	N-terminal EF-hand calcium binding protein 3	-0.39	0.95
SLC6A17	solute carrier family 6, member 17	-0.22	0.95
LRFN2	leucine rich repeat and fibronectin type III domain containing 2	-0.25	0.95
FAM134A	family with sequence similarity 134, member A	-0.25	0.95
C11orf57	chromosome 11 open reading frame 57	-0.28	0.94
CACNB1	calcium channel, voltage-dependent, beta 1 subunit	-0.08	0.94
NIPA1	non imprinted in Prader-Willi/Angelman syndrome 1	-0.18	0.94
GTPBP2	GTP binding protein 2	-0.39	0.94
EBF4	early B-cell factor 4	-0.24	0.94
CDR2L	cerebellar degeneration-related protein 2-like	-0.2	0.94
ETV6	ets variant gene 6 (TEL oncogene)	-0.31	0.94
RAPGEFL1	Rap guanine nucleotide exchange factor (GEF)-like 1	-0.22	0.94
CGREF1	cell growth regulator with EF-hand domain 1	-0.35	0.94
CCNJ	cyclin J	-0.46	0.94
KCNIP3	Kv channel interacting protein 3, calsenilin	-0.25	0.94
PSCD1	pleckstrin homology, Sec7 and coiled-coil domains 1(cytohesin 1)	-0.3	0.93
CTF8	chromosome transmission fidelity factor 8 homolog (S. cerevisiae)	-0.38	0.93
MSI1	musashi homolog 1 (Drosophila)	-0.21	0.93
TAF9B	TAF9B RNA polymerase II, TATA box binding protein (TBP)-associated factor, 31kDa	-0.39	0.93
SGPL1	sphingosine-1-phosphate lyase 1	-0.25	0.93
TMPRSS13	transmembrane protease, serine 13	-0.31	0.93
SEMA4C	sema domain, immunoglobulin domain (Ig), transmembrane domain (TM) and short cytoplasmic domain, (semaphorin) 4C	-0.28	0.93
CDH5	cadherin 5, type 2, VE-cadherin (vascular epithelium)	-0.43	0.93
ABHD6	abhydrolase domain containing 6	-0.31	0.93
ENTPD4	ectonucleoside triphosphate diphosphohydrolase 4	-0.22	0.92
ZFYVE1	zinc finger, FYVE domain containing 1	-0.37	0.92
MTUS1	mitochondrial tumor suppressor 1	-0.23	0.92
ATXN1	ataxin 1	-0.19	0.92
LNPEP	leucyl/cystinyl aminopeptidase	-0.25	0.92
DIRAS1	DIRAS family, GTP-binding RAS-like 1	-0.3	0.92
ESRRA	estrogen-related receptor alpha	-0.37	0.92
SH3BP4	SH3-domain binding protein 4	-0.28	0.92
ICHTHYIN	ichthyin protein	-0.51	0.92
SUV39H1	suppressor of variegation 3-9 homolog 1 (Drosophila)	-0.3	0.92
MTMR3	myotubularin related protein 3	-0.2	0.92
FAM118A	family with sequence similarity 118, member A	-0.27	0.92

Target	Full Name	Context Score	P_{CT}
RBM38	RNA binding motif protein 38	-0.2	0.92
EIF5A2	eukaryotic translation initiation factor 5A2	-0.15	0.92
FAM116A	family with sequence similarity 116, member A	-0.33	0.92
CDC42SE1	CDC42 small effector 1	-0.29	0.92
HCN3	hyperpolarization activated cyclic nucleotide-gated potassium channel 3	-0.34	0.92
USP37	ubiquitin specific peptidase 37	-0.19	0.91
KIAA0644	KIAA0644 gene product	-0.33	0.91
FUT4	fucosyltransferase 4 (alpha (1,3) fucosyltransferase, myeloid-specific)	-0.65	0.91
RND2	Rho family GTPase 2	-0.31	0.91
SOX11	SRY (sex determining region Y)-box 11	-0.41	0.91
FBXO45	F-box protein 45	-0.29	0.91
PCSK7	proprotein convertase subtilisin/kexin type 7	-0.28	0.91
TP53INP1	tumor protein p53 inducible nuclear protein 1	-0.29	0.91
MCL1	myeloid cell leukemia sequence 1 (BCL2-related)	-0.42	0.91
EAF1	ELL associated factor 1	-0.12	0.91
NFIB	nuclear factor I/B	-0.09	0.91
LIFR	leukemia inhibitory factor receptor alpha	-0.28	0.91
MAN1B1	mannosidase, alpha, class 1B, member 1	-0.3	0.91
NEU1	sialidase 1 (lysosomal sialidase)	-0.4	0.91
BAK1	BCL2-antagonist/killer 1	-0.56	0.9
SLC35A4	solute carrier family 35, member A4	-0.34	0.9
KIAA1244	KIAA1244	-0.18	0.9
MAP2K7	mitogen-activated protein kinase kinase 7	-0.26	0.9
VDR	vitamin D (1,25- dihydroxyvitamin D3) receptor	-0.26	0.9
SLC39A13	solute carrier family 39 (zinc transporter), member 13	-0.24	0.9

Supplemental Material, Table S2: Transcriptional targets predicted to be regulated by miR-142-3p. P_{CT} refers to the probability of preferentially conserved targeting.

Target	Full Name	Context Score	P_{CT}
ASH1L	ash1 (absent, small, or homeotic)-like (Drosophila)	-0.59	> 0.99
RICTOR	rapamycin-insensitive companion of mTOR	-0.57	> 0.99
ITGB8	integrin, beta 8	-0.62	0.98
C20orf194	chromosome 20 open reading frame 194	-0.72	0.97
SNF1LK	SNF1-like kinase	-0.39	0.97
FAM44B	family with sequence similarity 44, member B	-0.65	0.95
C10orf18	chromosome 10 open reading frame 18	-0.87	0.95
ZCCHC14	zinc finger, CCHC domain containing 14	-0.5	0.94
AFF1	AF4/FMR2 family, member 1	-0.25	0.94
EML4	echinoderm microtubule associated protein like 4	-0.42	0.93
LCOR	ligand dependent nuclear receptor corepressor	-0.33	0.93
CCDC6	coiled-coil domain containing 6	-0.33	0.92
BNC2	basonuclin 2	-0.32	0.9

Supplemental Material, Table S3: Pathways significantly associated with the predicted targets of miR-142-3p.

--

Canonical Pathways	p-value	miR-142-3p Predicted Targets
ILK Signaling	0.008	*ITGB8, RICTOR*
Role of IL-17F in Allergic Inflammatory Airway Diseases	0.031	*SIK1*
Macropinocytosis Signaling	0.048	*ITGB8*

Supplemental Material, Figure S1: Predicted mRNA targets of formaldehyde-altered miR-125b are involved in apoptosis signaling. The apoptosis signaling pathway is illustrated, where molecules predicted to be targeted by miR-125b are shaded in dark grey.

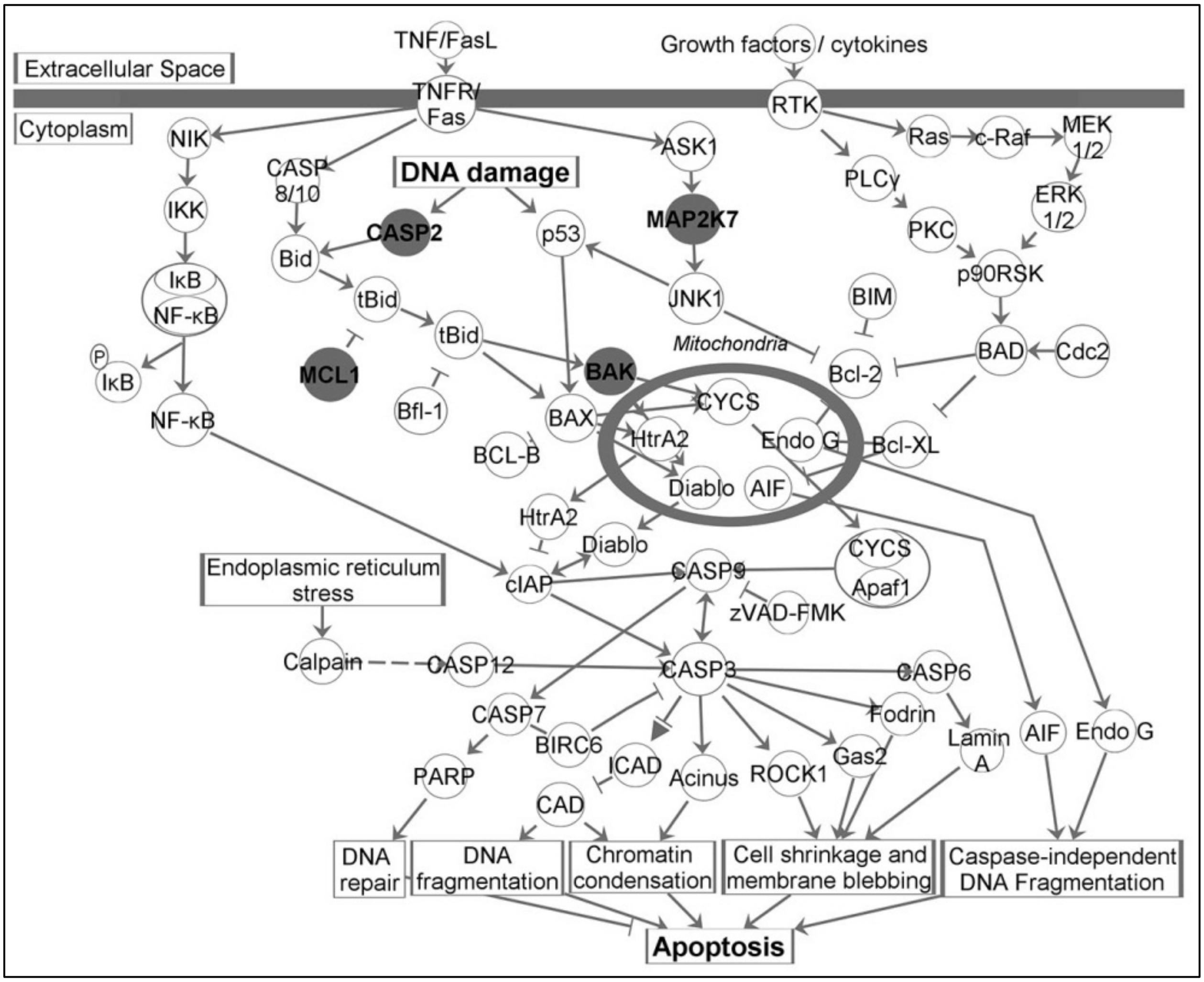

Supplement: (696 KB) PDF [file ehp.1205582.s001.pdf]
